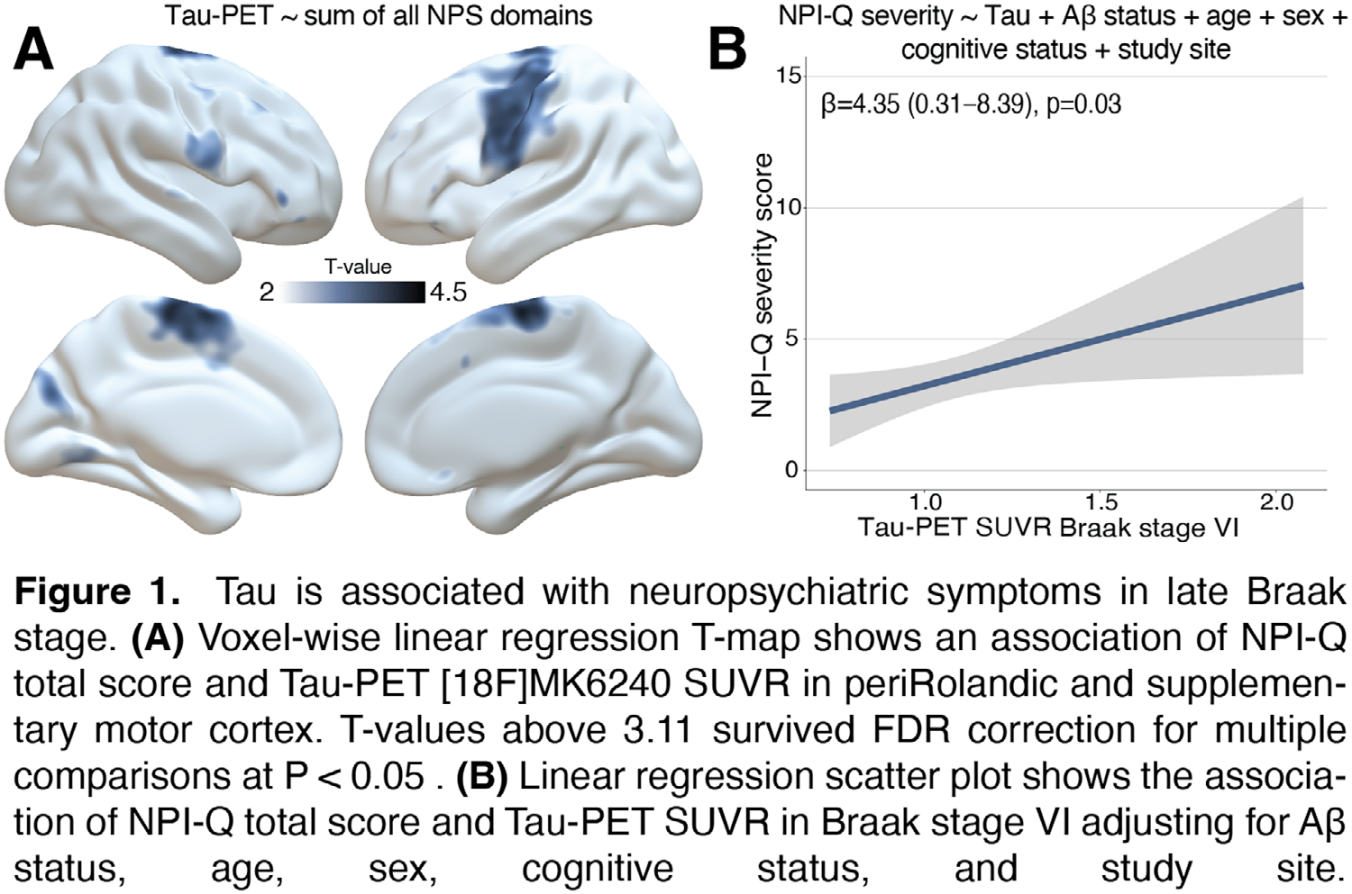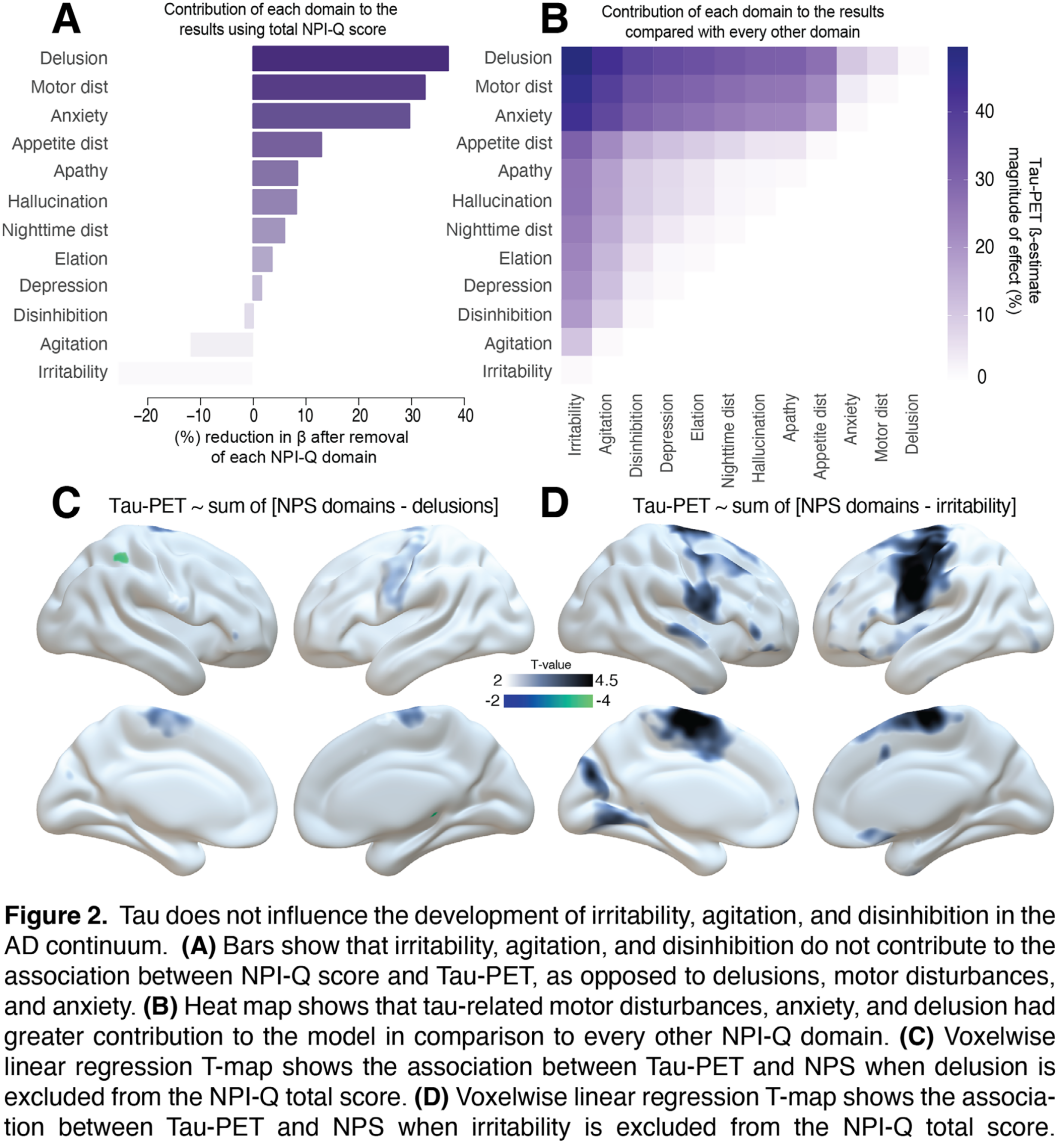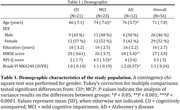# Tau exacerbates the development of distinct neuropsychiatric symptoms in late braak stage – the head study

**DOI:** 10.1002/alz.092310

**Published:** 2025-01-03

**Authors:** Cristiano Schaffer Aguzzoli, Guilherme Povala, Pamela C.L. Ferreira, Firoza Z Lussier, Guilherme Bauer‐Negrini, Livia Amaral, Carolina Soares, João Pedro Ferrari‐Souza, Hussein Zalzale, Bruna Bellaver, Francieli Rohden, Sarah Abbas, Douglas Teixeira Leffa, Lucas Porcello Schilling, Eduardo R. Zimmer, Dana Tudorascu, Belen Pascual, Brian A. Gordon, Val J. Lowe, Hwamee Oh, David N. Soleimani‐Meigooni, Pedro Rosa‐Neto, Suzanne L. Baker, Tharick Ali Pascoal

**Affiliations:** ^1^ Brain Institute of Rio Grande do Sul, PUCRS, Porto Alegre, RS Brazil; ^2^ Global Brain Health Institute, San Francisco, CA USA; ^3^ University of Pittsburgh, Pittsburgh, PA USA; ^4^ Universidade Federal do Rio Grande do Sul, Porto Alegre, Rio Grande do Sul Brazil; ^5^ Universidade Federal do Rio Grande do Sul, Porto Alegre Brazil; ^6^ Houston Methodist Research Institute, Houston, TX USA; ^7^ Washington University in St. Louis School of Medicine, St. Louis, MO USA; ^8^ Department of Radiology, Mayo Clinic, Rochester, MN USA; ^9^ Brown University, Providence, RI USA; ^10^ Memory and Aging Center, Weill Institute for Neurosciences, University of California, San Francisco, San Francisco, CA USA; ^11^ Translational Neuroimaging Laboratory, The McGill University Research Centre for Studies in Aging, Montréal, QC Canada; ^12^ Lawrence Berkeley National Laboratory, Berkeley, CA USA

## Abstract

**Background:**

Recent studies showed that neuroinflammation plays a key role in triggering specific neuropsychiatric symptoms (NPS), such as irritability and agitation, in individuals with Alzheimer’s disease (AD). While prior studies showed an association between tau pathology and all NPS domains, the extent to which tau influences each specific NPS domain remains unclear. Here, we aim to investigate the association of tau and NPS domains in the AD continuum. We hypothesize that tau plays a comparatively greater effect on the emergence of psychotic symptoms compared to other NPS domains.

**Method:**

We assessed 56 individuals (21 cognitively unimpaired (CU), 23 MCI, and 12 AD dementia) from the HEAD study who underwent clinical assessments with the Neuropsychiatry Inventory Questionnaire (NPI‐Q) and had positron emission tomography (PET) for amyloid‐β (Aβ) ([^18^F]AZD4694 or [^11^C]PiB), and tau tangles ([^18^F]MK6240) at the same visit. We selected individuals with an NPI‐Q total score ≥1. Tau SUVR values were tailored with a mask from Braak stages I‐VI, using the inferior cerebellar gray matter as reference region. Leave‐one‐out voxel wise and linear regression tested the association between each NPI‐Q domain and biomarkers accounting for age, sex, cognitive status, and study site.

**Result:**

CI individuals had significantly higher NPI‐Q score and Braak VI PET SUVR than CU individuals (**Table 1**). NPI‐Q score was significantly associated with tau‐PET in the periRolandic and supplementary motor cortex (**Figures 1A**). Linear regression showed that NPI‐Q associates with tau‐PET in the Braak stage VI **(Figure 1B**). Leave‐one‐out regression analysis revealed that delusions, motor disturbances, and anxiety contributed most to the association between tau‐PET and NPS (**Figure 2A, C**). These domains presented a higher magnitude of association compared to each other NPI‐Q domain (**Figure 2B**). Notably, irritability, agitation, and disinhibition exerted a negative effect to the association, emphasizing that tau may not play a role in the development of these symptoms (**Figure 2A, D**)

**Conclusion:**

Our study supports previous evidence suggesting that irritability and agitation may not be triggered by tau, but rather by other pathological process such as neuroinflammation. These findings provide additional rationale for the therapeutics aiming to mitigate irritability and agitation in AD patients.